# 肿瘤相关巨噬细胞在肺癌中的研究进展

**DOI:** 10.3779/j.issn.1009-3419.2021.102.49

**Published:** 2022-01-20

**Authors:** 艳艳 乔, 恩清 傅

**Affiliations:** 710038 西安，空军军医大学第二附属医院呼吸与危重症医学科 Department of Respiratory and Critical Care Medicine, The Second Affiliated Hospital of Air Force Military Medical University, Xi'an 710038, China

**Keywords:** 肺肿瘤, 肿瘤相关巨噬细胞, 肿瘤微环境, Lung neoplasms, Tumor-associated macrophages, Tumor microenvironment

## Abstract

肺癌是全球发病率和死亡率最高的恶性肿瘤之一。因此对于肺癌治疗手段的研究也在不断深入，目前临床上主要有全身化疗、针对驱动基因阳性的靶向治疗、免疫检查点抑制剂的应用、抗肿瘤血管生成治疗以及上述不同治疗方法的联合等，这些方案的使用明显改善了大多数肺癌患者的预后，但晚期患者预后仍然不尽如人意。近年来，与免疫相关的肿瘤微环境（tumor microenvironment, TME）的研究越来越受到重视。TME由免疫细胞、成纤维细胞、血管内皮细胞等细胞成分及相关的细胞因子等组成，是肿瘤细胞赖以生存、发展的基础。而肿瘤相关巨噬细胞（tumor-associated macrophages, TAMs）是TME重要的免疫细胞，指浸润于肿瘤组织中的巨噬细胞，可促进肿瘤细胞增殖，诱导肿瘤免疫耐受，刺激肿瘤血管生成，增加肿瘤细胞的侵袭及转移能力。因此，靶向TAMs已经成为肺癌免疫治疗的热点。本文就TAMs来源、表型及其在肺癌中的作用机制以及在未来治疗中的靶点进行综述，为肺癌最优化治疗提供参考。

## Abstract (en)

Lung cancer is one of the malignant tumors with the highest morbidity and mortality in China. Therefore, the research on the treatment of lung cancer is also deepening. At present, there are mainly systemic chemotherapy, targeted therapy for positive driver genes, the application of immune checkpoint inhibitors, anti-tumor angiogenesis therapy and the combination of the different treatment methods mentioned above. The use of these regimens has significantly improved the prognosis of most lung cancer patients, but the prognosis of patients with advanced lung cancer remains unsatisfactory. Recently, more and more attention has been paid to the study of tumor microenvironment (TME). TME consists of immune cells, fibroblasts, vascular endothelial cells and other cellular components as well as related cytokines, which is the basis for the survival and development of tumor cells. As an important immune cell of TME, tumor-associated macrophages (TAMs) refer to macrophages infiltrating in tumor tissues, which can promote tumor cell proliferation, induce tumor immune tolerance, stimulate tumor angiogenesis, and increase the invasion and metastasis ability of tumor cells. Therefore, targeting TAMs has become a hot topic in lung cancer immunotherapy. In this review, the sources, phenotypes, mechanisms of TAMs in lung cancer, as well as future therapeutic targets of TAMs were reviewed to provide reference for optimal treatment of lung cancer.

目前世界上肺癌的发病率呈现不断上升的趋势。最新的流行病学调查表明，在男性中肺癌是发病率最高的恶性肿瘤，在女性中其发病率仅次于乳腺癌，但仍是癌症相关死亡的最常见原因^[1]^。以往对于肺癌的治疗人们多关注于肿瘤细胞自身，随着医学研究的深入，肿瘤微环境在肺癌中的作用逐渐引起了人们的关注。肿瘤微环境（tumor microenvironment, TME）由免疫细胞、成纤维细胞及相关的细胞因子等组成，为肿瘤的发生发展提供了必要的条件，其中巨噬细胞在肿瘤微环境中发挥了重要作用^[2, 3]^。肿瘤相关巨噬细胞（tumor-associated macrophages, TAMs）是指参与构成肿瘤微环境的巨噬细胞，影响肺癌发生发展全过程。本文就TAMs在肺癌中的作用以及未来治疗策略中相关靶点进行综述。

## 1 肺巨噬细胞与TAMs

巨噬细胞具有多样性和高度异质性，在人体绝大部分器官组织中均有分布，且执行不同的功能。一直以来人们普遍认为巨噬细胞来源于骨髓造血干细胞，并在外周血中分化为单核细胞，其一旦进入局部组织就会转变为支持局部组织功能的组织特异性巨噬细胞，其中包括进入肺内转变为肺巨噬细胞。但目前有研究表明肺巨噬细胞不只来源于骨髓造血干细胞。根据来源不同肺巨噬细胞分为组织特异的巨噬细胞（tissue-resident macrophages, TRMs）和单核细胞来源的巨噬细胞（monocyte-derived macrophages, MDMs），TRMs在人出生前已经存在，可独立于成年造血系统，并在局部进行自我更新，协调组织重塑和维持组织完整性^[4]^。MDMs来自成人造血干细胞，组织发生炎症病变时可见其大量聚集于病变部位^[5]^。同时在研究肿瘤微环境中也发现有这两类TAMs，但其功能不同。MDMs在肿瘤微环境中抑制肿瘤生长，TRMs在组织内稳态和宿主防御中起重要作用。而近期Casanova-Acebes等^[6]^研究发现，在人类和小鼠非小细胞肺癌（non-small cell lung cancer, NSCLC）病程早期就有TRMs，更确切的讲即肿瘤相关肺泡巨噬细胞，聚集在肺癌细胞附近，促进癌细胞侵袭，同时诱导调节性T细胞（regulatory T cell, Treg）增多来促进肿瘤免疫逃逸。而去除TRMs可减少Treg的数量，并促使CD8^+^ T细胞的聚集，抑制肿瘤的生长。同时发现，小鼠肺中单核细胞来源的巨噬细胞有促进肿瘤转移作用，而组织内的巨噬细胞被发现与肿瘤生长相关^[7]^。综上所述，可以发现虽然TRMs和MDMs来源不同，但从不同方面促进了肺癌生长、转移。此外在肺癌小鼠模型中发现，单核细胞来源的巨噬细胞和组织特异的巨噬细胞均被证实为TAMs^[8]^。

在健康肺中，TRMs按照解剖部位不同分为肺泡巨噬细胞（lung alveolar macrophages, AMs）和间质巨噬细胞（interstitial macrophages, IMs）。AMs主要位于肺泡腔。IMs主要位于支气管血管束间质中，IMs由几个亚群组成^[9]^。AMs起源于卵黄囊，但随着时间的推移，被胎儿肝单核细胞取代，高度依赖于粒细胞-巨噬细胞集落刺激因子（granulocyte-macrophage colony stimulating factor, GM-CSF）而发展为成熟的AMs^[10]^。胚胎来源的AMs终生自我维持，而IMs则由循环中的单核细胞缓慢补充。以往认为两者具有不同的起源而无法进行相互转化。新的证据表明肺间质巨噬细胞来源于胚胎，并可由骨髓来源的循环单核细胞补充^[11]^。目前AMs和IMs在肿瘤微环境中的作用尚不十分清楚，在生成TAMs中的作用有待进一步研究。

## 2 TAMs表型与功能

TAMs的可塑性极强，表现为在不同微环境刺激下极化为不同的表型、执行不同的功能。根据功能及表型不同，TAMs主要分为经典激活的M1型和替代激活的M2型巨噬细胞^[12]^。在肺癌中TAMs常表现为M2型巨噬细胞的作用。

激活M1型巨噬细胞的因子有：GM-CSF或集落刺激因子-2（colony stimulating factor 2, CSF-2）和促炎刺激因子如肿瘤坏死因-*α*（tumor necrosis factor-*α*, TNF-*α*）、*γ*干扰素（interferon *γ*, IFN-*γ*）、脂多糖（lipopolysaccharides, LPS）。LPS或IFN-*γ*等细胞因子激活相关通路，其中最主要的是通过ICAM1-PI3K-Akt-Notch1及JAK1-STAT1-Caspase通路^[13]^，以上调M1型巨噬细胞相关基因表达，促使M1型巨噬细胞活化，而活化后的M1型巨噬细胞分泌较多的IL-6、TNF-*α*、白介素12（interleukin-12, IL-12）和活性氧，参与辅助性Th1细胞对感染的应答，以促进炎症和抵御病原体，并抑制肺癌生长甚至促使肿瘤细胞凋亡^[14]^。

激活M2型巨噬细胞的因子包括：集落刺激因子-1（colony stimulating factor 1, CSF-1）和IL-4、IL-13和IL-10。其中，IL-4和IL-13通过IL-4受体*α*激活STAT6通路^[15]^，促使M2型巨噬细胞活化增多。此外，其他细胞因子如IL-10也可以通过IL-10受体激活STAT3来调控向M2型巨噬细胞极化^[16]^。活化后的M2型巨噬细胞分泌多种抗炎细胞因子，如转化生长因子-β（transforming growth factor-β, TGF-β）、IL-10和精氨酸酶等^[17]^，这些因子进一步加速TME的重塑，并分别通过多种机制促进肿瘤的生存、发展和转移^[18]^。

M1和M2型巨噬细胞有明显的功能差异，根据其所受环境刺激因子不同可相互转化。研究^[19]^表明，在肺癌早期以M1型巨噬细胞为主，而中晚期则以M2型巨噬细胞为主，随着肿瘤进展，M1逐渐向M2表型转化。在我国肺癌一经确诊，大多数进入晚期，因此相关研究也发现在肺癌中以分化为M2型为主。同时研究^[20]^发现巨噬细胞可严重浸润肺癌组织，其占肿瘤体积高达50%，进一步证实了其在促进肿瘤进展过程中的重要作用。此外，M2型巨噬细胞还可以释放表皮细胞生长因子（epidermal growth factor, EGF）等细胞因子促进癌细胞的转移^[21]^。最后研究^[22]^还表明，在肺癌中，M2型巨噬细胞浸润严重的患者预后更差、总生存率更低。

## 3 TAMs在肺癌发病机制中的作用

早在19世纪，炎症和癌症之间的联系就被发现，当时德国病理学家Rudolf Virchow首次证明肿瘤中存在白细胞^[23]^。随后经过大量动物实验及临床数据证明，肿瘤发生的所有阶段（包括起始到转移一系列步骤）都受到了肿瘤相关炎症的影响^[24]^。同样，肺恶性肿瘤也与炎症有关。巨噬细胞也参与肺恶性肿瘤生长、发展等全部过程，开始于早期侵袭前阶段，肿瘤细胞通过释放细胞因子和外泌体，将巨噬细胞等吸引到肿瘤间质中^[25]^，同时M2型巨噬细胞分泌多种细胞因子等促进肿瘤生长、侵袭和转移^[26]^。

### 3.1 TAMs促进肺癌的侵袭、转移

大量研究证实，在肺癌中，TAMs通过产生各种趋化因子和细胞因子促进肿瘤侵袭和转移，其主要分泌包括TGF-β、IL-10、IL-6、基质金属蛋白酶（matrix metalloproteinases, MMPs）、血管内皮生长因子（vascular endothelial growth factor, VEGF）等。

TAMs可能通过分泌IL-10激活肿瘤干细胞以促进肿瘤进展^[27]^。当肿瘤细胞过快生长时，就会出现缺氧。在Lewis小鼠肺癌细胞模型中发现，缺氧促使M2型巨噬细胞生成增多，同时上调IL-10、VEGF和低氧诱导因子-1*α*（hypoxia inducible factor-1*α*, HIF-1*α*）增加，进一步促进癌细胞转移，并募集更多巨噬细胞浸润原发肿瘤组织^[28]^。

上皮-间充质转化（epithelial mesenchymal transition, EMT）是指上皮细胞失去极性，具有了间充质细胞的特性。EMT在NSCLC转移中起重要作用^[29]^，但其潜在的分子机制尚不十分清楚。TAMs释放IL-6、IL-10、TGF-β等细胞因子，这些细胞因子均可调节EMT^[30]^，此外，IL-6、IL-10也可通过激活JAK/STAT3以IL-4依赖的方式诱导M2型巨噬细胞分化^[31]^，TGF-β可通过C-jun/SMAD3途径促进SOX9的表达，增强肺癌细胞增殖、侵袭和转移能力^[32]^。

最后，TAMs分泌MMPs，主要包括MMP-9和MMP-2，以降解细胞外基质，促侵袭、转移^[33]^。此外，MMP-9表达与淋巴结转移、预后相关^[34]^。

### 3.2 TAMs抑制抗肿瘤免疫TAMs抑制抗肿瘤免疫

机制如下：①在TME中，TAMs不仅失去了抗肿瘤特性，还阻碍其他免疫细胞的免疫调节功能。由Th2细胞刺激的M2型巨噬细胞在肺TME中产生免疫抑制因子如IL-10和TGF-β，其中IL-10通过上调肿瘤巨噬细胞中PD-L1的表达以抑制细胞毒性T细胞功能，导致免疫耐受^[35]^。②TAMs释放的CC类趋化因子配体22（C-C motif ligand 22, CCL-22）通过招募Tregs至TME中，抑制效应T细胞的活化和功能^[36]^，产生免疫抑制的微环境，诱导免疫耐受。③当肿瘤细胞增殖不受控制时，氧气和营养受到限制，进而导致缺氧。缺氧一方面增强巨噬细胞HIF-1*α*的表达导致巨噬细胞介导的CD8^+^ T细胞活化减少，促使了免疫逃逸^[37]^。另一方面，缺氧时，TAMs产生的精氨酸酶I增多，其将微环境中L-精氨酸耗尽，从而抑制T细胞使其处于细胞周期的G_0_期、G_1_期而无法增殖^[38]^。④TAMs表面表达的CD206甘露糖受体通过抑制CD45磷酸酶活性，从而导致细胞毒性T淋巴细胞相关蛋白4（cytotoxic T lymphocyte-associated antigen-4, CTLA-4）上调，最终诱导T细胞耐受^[39]^。⑤最后，已有研究^[40]^表明IL-4刺激激活巨噬细胞PI3K*γ*-mTor-S6K*α*-C/EBPβ通路和抑制核因子κB（nuclear factor kappa-B, NF-κB）来抑制免疫，促进了肿瘤的生长。

### 3.3 TAMs促进肿瘤血管生成

因为肿瘤细胞常常不受控制的增殖，容易出现缺氧和酸性微环境，因此血液供应并输送氧气和营养成分、清除代谢废物至关重要^[41]^。研究^[42]^表明在TME中，TAMs是调控血管生成的关键细胞。TAMs常分泌VEGF，不仅诱导新生血管形成，而且增加血管渗透性和调控肿瘤细胞的血管内扩散，导致远处转移^[43]^。此外，TAMs还可以分泌TGF-β、PDGF、TNF-*α*、MMPs和EGF等多种促血管生成因子以促进肿瘤内血管的形成。缺氧是肿瘤血管生成的主要驱动条件^[44]^。在低氧情况下，TAMs表达转录因子HIF-1*α*，诱导VEGF、血小板衍生生长因子（platelet derived growth factor, PDGF）、EGF等转录来促进血管生成^[45]^。

## 4 有关TAMs潜在治疗靶点

通过对肺癌深入研究，人们发现TAMs对肺癌的发生发展产生了很大影响。因此，针对TAMs的靶向治疗策略，可能会增强肺癌全身化疗、免疫治疗等的疗效^[46]^。目前针对巨噬细胞的潜在治疗方法正在探索及研究中，以下3条途径可能抑制肺癌进展：改变TAMs表型、阻止TAMs募集、耗竭TAMs^[47]^。

### 4.1 改变TAMs表型

肺癌中M2型巨噬细胞密度与生存恶化相关^[48]^。M1型巨噬细胞密度与较高的总生存率相关^[22]^。因此重编程或巨噬细胞再极化为抗肿瘤表型是肿瘤免疫治疗的重要研究方向。如前所述，可塑性是巨噬细胞的关键特性，在某种特定的微环境或信号诱导下可以使其改变自身的表型。因此，控制极化平衡的分子靶点被认为是癌症治疗的重要途径。

研究^[49]^发现CD40信号参与单核细胞分化为M1型巨噬细胞，并可将M2型巨噬细胞逆转为M1型。因此靶向CD40/CD40L成为了肿瘤免疫治疗研究热点。CD40靶向激动剂单克隆抗体联合化疗或免疫检查点抑制剂已经在NSCLC中开始了初步临床试验，有望尽早用于临床。

TAMs受体抑制剂是逆转M2型巨噬细胞向M1型转化的另一治疗靶点。TAMs受体，包括Tyro3、Axl和MERTK，是酪氨酸激酶受体家族，共享配体Gas6和Protein S，使巨噬细胞向M2样表型极化^[50]^。研究^[51, 52]^报道NSCLC中过表达Axl和MERTK。同样研究^[53]^证实了MERTK小分子抑制剂（如UNC2025）降低小鼠NSCLC模型中肿瘤远处转移。

在Lewis小鼠肺癌中，伊马替尼通过抑制STAT6磷酸化和核易位，抑制巨噬细胞M2样的极化，可以预防肺癌转移，为肺癌的治疗提供新选择^[54]^。有望大规模临床试验，甚至多中心RCT研究以惠及更多患者。

最后的治疗方法是阻断M2型巨噬细胞的激活分子，目前研究表明，这种方法可以提高免疫检查点抑制剂等疗法的效果。针对促使极化为M2型巨噬细胞细胞因子如IL-13、IL-4和IL-10来研发免疫抑制药物，可有效治疗肿瘤中促瘤型TAMs亚型^[55]^。IL-10与M2型巨噬细胞密切相关，据相关报道^[56]^，TAMs中高表达的IL-10与NSCLC分期相关。关于这些细胞因子的研究在未来很可能成为肺癌靶向治疗新的研究方向。

### 4.2 阻止TAMs募集

在肿瘤发展、新生血管形成和转移中，TAMs起着重要作用。减少肿瘤微环境中TAMs的数量是通过减少循环单核细胞来的补充。参与募集的因素包括：趋化因子（如CCL2）、细胞因子（如CSF-1）和补体介质等，因此针对这些趋化因子及其受体、细胞因子或补体介质的调控来抑制TAMs募集是未来研究的方向。目前研究最深入的有CCL2-CCR2、CSF-1和CSF-1R通路。

CCL2-CCR2信号已被证明能够募集TAMs来激发肿瘤新生血管形成、促进癌细胞快速增长^[57]^。在Lewis小鼠肺癌模型研究中发现，去除CCR2基因或使用CCR2抑制剂（RS504393），均抑制TAMs募集并促使TAMs极化为M1型，同时抑制肺癌血管生长，抑制肺癌进展^[58]^。

抑制TAMs向肿瘤微环境募集的另一个途径是抑制CSF1-CSF1R轴，这对TAMs的分化、存活和招募都很重要^[59]^。CSF-1R阻断剂可以减少抑制性T细胞介导的TAMs的浸润^[60]^。在动物模型中，已经证实小分子CSF-1R抑制剂与免疫检查点抑制剂联合，用于治疗晚期乳腺癌等实体肿瘤正处于I期/II期临床试验中^[61]^，这一发现对于靶向抑制肺癌TAMs募集提供了新思路。

### 4.3 耗竭TAMs

多项研究证实，TAMs在肿瘤组织中的密度与预后不良有关，密度越高肿瘤生长越快，因此对于已经存在于TME中的TAMs消耗殆尽是抑制肿瘤生长的有效方法。早期研究^[62]^发现双膦酸盐可以清除单核细胞和巨噬细胞。目前研究最深入的耗尽TAMs是抑制CSF-1/CSF-1R信号轴，如前所述目前已研发出许多策略来干扰这一巨噬细胞生存途径，包括针对CSF-1或CSF-1R小分子抑制剂的单克隆抗体。研究^[63]^表明CSF-1R表达于肿瘤微环境中TAMs上，CSF-1R抑制剂可能会在肿瘤微环境中耗尽M2型巨噬细胞，Emactuzumab（NCT02323191）是针对CSF-1R单克隆抗体，与免疫治疗药物联合治疗已用于NSCLC临床试验。

TAMs的耗竭也可以通过靶向表面分子来实现，包括CD52、清道夫受体A（scavenger receptor-A, SR-A）、叶酸受体β（folic acid receptor-β, FR-β）和CD206^[64]^。通过对这些靶点的干预均可耗尽促肿瘤巨噬细胞，从而抑制血管生成，延缓肿瘤进展，可能在预防肺癌进展中有一定的前景。

## 5 结语

综上所述，TAMs在肺癌的发展过程中起着重要作用，其极化状态、浸润严重程度与患者预后密切相关。因此，进一步阐明TAMs表型及其相关分子通路在肺癌发生发展的研究中具有重要意义，可为肺癌的免疫治疗提供新思路，为肿瘤新药研发提供重要的理论依据。但目前相关药物的研究正处于动物试验阶段或临床试验阶段，尚未在临床治疗中应用。因此针对TAMs的靶向疗法的研究或成为未来肺癌免疫治疗中的重要手段。
